# Conceptual and institutional gaps: understanding how the WHO can become a more effective cross-sectoral collaborator

**DOI:** 10.1186/s12992-015-0128-6

**Published:** 2015-11-24

**Authors:** Unni Gopinathan, Nicholas Watts, Daniel Hougendobler, Alex Lefebvre, Arthur Cheung, Steven J. Hoffman, John-Arne Røttingen

**Affiliations:** Institute of Health and Society, University of Oslo, Oslo, Norway; Institute of Global Health, University College London, London, UK; O’Neill Institute for National and Global Health Law, Georgetown University Law Center, Washington, DC USA; Faculty of Medicine, University of British Columbia, Vancouver, Canada; School of Medicine, The University of Queensland, Brisbane, Australia; Global Strategy Lab, Faculty of Law, University of Ottawa, Ottawa, Canada; Department of Global Health & Population, Harvard T.H. Chan School of Public Health, Harvard University, Boston, MA USA; Department of Clinical Epidemiology & Biostatistics and McMaster Health Forum, McMaster University, Hamilton, Canada; Division of Environmental Health and Infectious Disease Control, Norwegian Institute of Public Health, Oslo, Norway

**Keywords:** World Health Organization, United Nations, Global governance, Global health governance, Global governance for health, Social determinants of health, Health in all policies, WHO reform, Cross-sectoral collaboration

## Abstract

**Background:**

Two themes consistently emerge from the broad range of academics, policymakers and opinion leaders who have proposed changes to the World Health Organization (WHO): that reform efforts are too slow, and that they do too little to strengthen WHO’s capacity to facilitate cross-sectoral collaboration. This study seeks to identify possible explanations for the challenges WHO faces in addressing the broader determinants of health, and the potential opportunities for working across sectors.

**Methods:**

This qualitative study used a mixed methods approach of semi-structured interviews and document review. Five interviewees were selected by stratified purposive sampling within a sampling frame of approximately 45 potential interviewees, and a targeted document review was conducted. All interviewees were senior WHO staff at the department director level or above. Thematic analysis was used to analyze data from interview transcripts, field notes, and the document review, and data coded during the analysis was analyzed against three central research questions. First, how does WHO conceptualize its mandate in global health? Second, what are the barriers and enablers to enhancing cross-sectoral collaboration between WHO and other intergovernmental organizations? Third, how do the dominant conceptual frames and the identified barriers and enablers to cross-sectoral collaboration interact?

**Results:**

Analysis of the interviews and documents revealed three main themes: 1) WHO’s role must evolve to meet the global challenges and societal changes of the 21st century; 2) WHO’s cross-sectoral engagement is hampered *internally* by a dominant biomedical view of health, and the prevailing institutions and incentives that entrench this view; and 3) WHO’s cross-sectoral engagement is hampered *externally* by siloed areas of focus for each intergovernmental organization, and the lack of adequate conceptual frameworks and institutional mechanisms to facilitate engagement across siloes.

**Conclusion:**

There are a number of external and internal pressures on WHO which have created an organizational culture and operational structure that focuses on a narrow, technical approach to global health, prioritizing disease-based, siloed interventions over more complex approaches that span sectors. The broader approach to promoting human health and wellbeing, which is conceptualized in WHO’s constitution, requires cultural and institutional changes for it to be fully implemented.

**Electronic supplementary material:**

The online version of this article (doi:10.1186/s12992-015-0128-6) contains supplementary material, which is available to authorized users.

## Background

As the world grows increasingly interdependent, the importance of global governance to advancing public health has intensified and become more complex. Among the many different definitions of global governance is the well-known and comprehensive one by Weiss and Thakur [1] which describe global governance as “the complex of formal and informal institutions, mechanisms, relationships, and processes between and among states, markets, citizens, and organisations, both intergovernmental and non-governmental, through which collective interests on the global plane are articulated, rights and obligations are established, and differences are mediated”. The complexity in global health is exacerbated by the fact that decisions made outside of the health sector have profound impacts on global health. The interaction between trade liberalization and the global rise of non-communicable diseases [2], the public health effects of climate change [3], the health impacts of global migration [4], and the impact of social determinants on individual health [5], all illustrate the broader determinants of health that must be addressed beyond the confines of the health sector. This realization have prompted the development of concepts such as “global health diplomacy” [6] and “global governance for health” [7]—describing why and how global governance systems outside the global health system should protect and promote people’s health. This study, building on previously described definitions [8–10], distinguish between “global governance for health” and “global health governance”. Global health governance mainly refer to the collaboration between and the coordination of international institutions, bilateral aid agencies, non-governmental organizations, philanthropic organizations and public-private partnerships (such as the Global Alliance for Vaccination and Immunization and the Global Fund to Fight AIDS, Tuberculosis and Malaria) whose processes and activities primarily aim to improve global health. In comparison, global governance for health is about “institutions and processes of global governance which do not necessarily have explicit health mandates, but have a direct and indirect health impact” [8], and how these global institutions and processes can better work to improve global health.

In this context, the World Health Organization (WHO), with its mandate as the directing and coordinating authority for health within the United Nations system [11], is the natural starting point for efforts to strengthen global governance for health. The WHO has undergone multiple reform attempts, with the most recent process starting in 2010 and continuing to this day [12, 13]. One important aspect of WHO’s reform agenda is moving the organization beyond its traditional technical focus to a more proactive role where the organization more effectively address the broader determinants of health through cross-sectoral collaboration. However, over the years, two themes consistently emerge from the broad range of academics, policymakers and opinion leaders who have analyzed WHO reforms: that the reforms are too slow, and that they do too little to strengthen WHO’s capacity to facilitate cross-sectoral collaboration [14–16].

To inform this part of WHO’s reform agenda, there is need for a clear understanding of how the organization perceives and interacts with other sectors at the inter-governmental level. The main challenges, of translating theoretical frameworks into real-world cross-sectoral collaboration, need to be identified and well understood. This qualitative mixed methods study aims to identify and explain challenges WHO faces in addressing the broader determinants of health, and the potential opportunities for enhancing cross-sectoral collaboration. Specifically, it investigates how WHO conceptualizes its mandate on global health, what factors enable or impede WHO’s efforts to engage with the intergovernmental organizations (IGOs) across sectors, and how the dominant conceptual frame interacts with these factors.

## Methods

This qualitative study used a mixed methods approach of semi-structured interviews and document review to explore three research questions:

1. How does WHO conceptualize its mandate on global health?
2. What are the barriers and enablers to enhancing cross-sectoral collaboration between WHO and other IGOs?
3. How do the dominant conceptual frame and the identified barriers and enablers to cross-sectoral collaboration interact?

### Semi-structured interviews

Sampling methodology for interviewee selection sought to balance the need for data saturation with feasibility. Five interviewees were selected by stratified purposive sampling within a sampling frame of 45 potential interviewees from the level of WHO department director level or above. General management, partnerships and regional office staff were excluded. No interviewees declined to participate, and informed consent was collected prior to conducting the interviews. The interviews were conducted between October 2012–February 2013. Prior to collecting the data, approval was received from the Data Protection Official for Research under the Norwegian Social Science Data Services (project no. 31093).

An interview schedule (Table 1) was developed for this study, and used for the semi-structured interviews. Compared to the three research questions, the questions in the interview schedule were phrased as more open-ended topics, with the aim of exploring a broader range of issues and avoid asking leading questions.

**Table 1:** Interview schedule used for the semi-structured interviews of senior WHO leaders

Introduction	
1. Briefly outline some of the global governance challenges that you encounter in your work. Do these have an impact on global health? How so?	
Framing Global Health	
2. In the day-to-day affairs of your organization, how is the advancement of global health conceptualised and framed in relation to the organization’s stated primary objects? [Can you provide any specific examples?]	
a. How do you persuade other sectors to engage with global health?	
Advancing Global Health through Global Governance	
3. What steps are taken by your organization to ensure that health is protected and promoted within its deliberations, policies and activities? Where and when in the planning, deliberation, and implementation processes of your organization is health considered?	
4. What are the major barriers to collaboration between your organization and a global governance institution from a different sector, towards the advancement of global health?	
5. What are the major enablers to collaboration between your organization and a global governance institution from a different sector, towards the advancement of global health?	
6. Given the major barriers and enablers, and the conceptual (2) and procedural (3) contexts discussed, are there any proposals or solutions you would like to see?	

All interviews were recorded and transcribed, and then anonymized to enable interviewees to express the fullest range of views and opinions without constraint. Field notes were also taken by interviewers, which were similarly anonymized.

### Document review

A document review was conducted to triangulate information and provide a point of reference for claims made during the interviews. A targeted review of strategic plans, financial statements, internal reform documents and external organization evaluations was conducted (see Additional file 1 for a complete list of documents reviewed).

Information was extracted from these documents using a data collection matrix designed to identify the dominant frames used to conceptualize global health, existing forms of collaboration with other sectors, and to corroborate the themes that emerged from the interviews (see Additional file 2 for the matrix). The development of the data collection matrix was informed by a literature review on framing in global health [17–25]. Frames used in the various papers were compared, and overlapping frames were defined under a common concept. The final data collection matrix contained eight frames (Table 2).

**Table 2:** Global health frames included in the data collection matrix for the document review

• Global health as biomedicine	
• Global health as a commodity/trade issue	
• Global health as foreign policy	
• Global health as a global public good	
• Global health as a human right	
• Global health as investing in economic growth	
• Global health as a means to reduce poverty	
• Global health as security	

### Thematic analysis

The interview transcripts, field notes, and public documents were qualitatively analyzed in NVivo 10 using thematic analysis as described by Yin [26]. Thematic analysis organizes and encodes the data according to themes emerging from the data set. Identified themes capture important aspects of the data in relation to the research question and facilitate the interpretation of the data set [26, 27]. Specifically, this study used free coding, iterative categorization of text fragments, and reciprocal translational analysis from meta-ethnography, integrating these techniques with grounded theory’s inductive approach and constant comparison method [28, 29].

To improve inter-rater reliability, both the analysis of semi-structured interviews and the review of WHO’s public documentation were performed by two independent investigators, who then compared their coding and discussed any reasons for variation. Interviews continued until data saturation was reached, as determined by two investigators who discussed the themes emerging from the interviews and whether new themes that addressed the research questions were still emerging. The data coded during the thematic analysis was analyzed against the three research questions.

## Results

Three major themes emerged from the interviews of senior WHO officials and the document review. The first theme describes that WHO’s role must evolve to meet global challenges and societal changes. The second and third themes are about the barriers to cross-sectoral collaboration. Common pressures experienced by organizations are often divided into factors external or internal to the organization, and the analysis identified this division to exist also for the barriers faced by the WHO. The second major theme describes that WHO’s cross-sectoral engagement is hampered internally by the dominant biomedical view of health, and the prevailing institutions and incentives that entrench this view. The third major theme is that WHO’s cross-sectoral engagement is hampered externally by siloed areas of focus for each IGO, and the lack of adequate conceptual frameworks and institutional mechanisms to facilitate engagement across siloes

### WHO’s role must evolve to meet the global challenges and societal changes of the 21st century

It was expressed that “WHO’s role as a convenor around global health issues” has undergone “a significant change”, and that now, “increasingly, everybody recognizes that health is part of a nexus of policy issues which affect trade, IP, the environment and etc.…” (Interviewee 3). As suggested by another interviewee below, there is an increasing need to consider the activities of a range of other sectors.

“…if we go back 3, 4 or 5 decades, a lot of the issues were very much focused on technical issues and the implementation of technical issues, how do you deal with smallpox?… but over time, it’s become clear that health itself is partly the result of a lot of activity going on in other sectors. It’s very hard to talk about health, without addressing poverty…It’s very hard to talk about the wellbeing of communities without looking at the development of communities. And it’s very hard to look at why countries should work together on health if you’re not looking at their foreign policy stance…”

It was furthermore raised that discussions on global health increasingly take the form of “political negotiations, rather than technical experts getting together” (Interviewee 3), and that health “is not any more negotiated by health officials mainly” (Interviewee 2). Overall, it was broadly agreed that addressing the various determinants of health requires the inclusion of a broader range of actors within a more complicated policy framework.

### WHO’s cross-sectoral engagement is hampered internally by the dominant biomedical view of health, and the prevailing institutions and incentives that entrench this view

The analysis of the interviews suggested that the operations of WHO and the global health system at large are characterized by a narrow, technical approach focusing on how the health sector can deal with diseases. Analysis of WHO’s strategic documents reiterated the dominance of this way of viewing global health challenges through a biomedical frame. We also identified three secondary frames that provide additional justification for WHO’s programmatic activities: global health as a human right, global health as a security issue, and global health as a means to reduce poverty (see Table 3). Finally, the strategic documents emphasized at different points the importance of social determinants of health, indicating the organization’s desire to view global health more broadly (Table 4).

**Table 3:** Prominent frames in the strategic documents of the World Health Organization

Global Health as biomedicine
The biomedical frame appears to be the dominant frame throughout almost all of WHO’s documents, which presents a disease-based conceptualisation of global health, with a focus on interventions within the health sector to reduce burden of specific diseases. Often, the structure of priorities or budget items are based almost exclusively on a biomedical frame, which extends to the very organization of WHO’s departments, which are dominantly structured according to specific disease groups.
Global health as a human rights issue
Health as a human right features prominently in the WHO constitution [11], which states that “the enjoyment of the highest attainable standard of health is one of the fundamental rights of every human being without distinction of race, religion, political belief, economic or social condition” Global health as a human right appears to be used as an additional justification for programmatic activities, almost always supplementing the biomedical frame. However, the programme budget for 2014–15 allocates very limited resources to the programme area on human rights [45].
Global health as a security issue
Global health as a security issue is a central and recurrent frame throughout many of WHO’s public documents, with an emphasis on WHO’s role in mitigating and coordinating international responses to disease outbreaks. This frame appears to be of particular importance to re-asserting the uniqueness and added value of WHO. However, it is notable that the security frame is less utilised in the GPW12, apart from references made to the International Health Regulations, and that the programme budget 2014–15 for outbreak and crisis response was cut by 51.4 % compared to the level in 2012–13 [45].
Global health as a means to reduce poverty
In the GPW11, eradicating extreme poverty is mentioned together with eradicating hunger as “the first and most important Millennium Development Goal”. While poverty reduction’s role in improving public health is acknowledged, the programme documents primarily frame the issue by discussing the role of health policies in contributing to poverty reduction. Indeed, ‘Investing in health to reduce poverty’ was one of the seven priorities for GPW11. The concept of ‘poverty reduction’ appears largely connected to the broader discussion on ‘sustainable development’ in the GPW12.
Rarely used frames
Global health as a global public good, global health as investing in economic growth, global health as foreign policy and global health as a commodity/trade issue were rarely used frames in the reviewed strategic documents.

**Table 4:** Examples of statements in strategic documents describing WHO’s role and barriers to addressing the broader determinants of health

Medium-term strategic plan 2008–2013
“Although essential for achieving lasting health improvements across populations, the underlying determinants of health have received relatively little attention at WHO, necessitating a substantial increase from the baseline.”
*“*lack of effective consensus among partners, including organizations of the United Nations system, other international bodies and nongovernmental organizations on policies and framework for action; insufficient investment by national governments for building and deploying adequate skills to ensure that tools to analyse human rights, ethical, economic, gender and poverty aspects are widely and effectively implemented.”
“The health sector is only poorly able to influence policies in other sectors to promote occupational and environmental health and lacks the tools, knowledge and skills to engage other sectors.”
“Health systems are on the whole not even identifying the environmental determinants of health as part of their remit, let alone as a priority for improving public health. The few existing data indicate that only about 2 % of a typical national health budget is currently invested in preventive health strategies. Clearly, health institutions face both the challenge of controlling health costs and the opportunity to do so through more effective environmental health strategies and interventions.”
“The mandate for WHO’s action in this area is firmly anchored in the Constitution and the history of public health practice and achievements. In the framework of United Nations reform, WHO has an opportunity to show a more global leadership in public health and the environment, linking health explicitly to the goals of sustainable development.”
Eleventh General Programme of Work 2006–2015
“Many of the determinants of health are outside WHO’s direct sphere of influence, but WHO will work with ministries of health to build their understanding of what can realistically be done by working with other sectors. WHO will monitor global trends that are of significance to health in areas such as trade and agriculture, and keep ministries of health informed.”
“More research is required for a better understanding of the links between determinants and their consequences, and for how governments, in particular ministries of health, can best influence other government sectors.”
“At the international level, governments will need to engage effectively with negotiated agreements such as TRIPS and the General Agreement on Trade in Services, given their increasing importance for health goods and services. Engagement with industry in general, covering areas such as food, pharmaceuticals and insurance, should continue, focusing on commonly agreed public health agendas. WHO has a responsibility to keep governments informed and engaged in the process.”
“The action required to tackle most of these determinants goes beyond the influence of ministries of health, and involves a large number of government and commercial responsibilities. If these determinants are to be dealt with effectively, therefore, the boundaries of public health action have to change. Governments, especially health ministries, must play a bigger role in formulating public policies to improve health, through collective action across many sectors. It is the responsibility of WHO to keep governments informed of the situation, raise awareness, and advocate policies to tackle the determinants when opportunities arise.”
Twelfth General Programme of Work 2014–2019
“The concept of social determinants of health constitutes an approach and a way of thinking about health that requires explicit recognition of the wide range of social, economic and other determinants associated with ill health, as well as with inequitable health outcomes. Its purpose is to improve health outcomes and increase healthy life expectancy. The wider application of this approach–in line with the title of the Twelfth General Programme of Work and in a range of different domains across the whole of WHO–is therefore a leadership priority for the next six years in its own right.”
“As a public health agency, WHO continues to be concerned not only with the purely medical aspects of illness, but with the determinants of ill health and the promotion of health as a positive outcome of policies in other sectors”
“One consequence of the growing political interest in health and the recognition of the connection between health and many other areas of social and economic policy, is a growing demand for intergovernmental, rather than purely technical processes, in order to reach durable and inclusive agreements. In the general programme of work it is foreseen that this demand is unlikely to decrease. As a consequence, WHO will put in place the requisite capacities to prepare for meetings, brief participants and manage these processes as effectively as possible.”

Two interviewees emphasized the importance of cross-sectoral engagement and negotiation in pursuit of public health benefits, and contrasted the biomedical frame with the importance of understanding the perspective of other sectors.

“Well, I think that it’s important to listen to what the other sectors have to say and not try to apply pre-cooked recipes, and then you have to see what flexibility there is. You cannot pretend that you will have ideal situations, sometimes you have to find compromise and the compromise needs to be good enough, so it’s a question of negotiation.” Interviewee 1

“[Lack of] capacity building in negotiation [is a barrier]. Or perhaps even, and that’s linked to attitude, realising that you need that capacity. You can’t take just a prescription and write on it, and [expect] the others are going to do [it]. Many health actors unfortunately are still in that illusion.” Interviewee 2

Interviewees noted that the health sector (including WHO) is often unable to speak effectively in the language and with the perspectives of other sectors, lacking the political savvy, communication skills, and relevant evidence to communicate the importance of health for the priorities and interests of other actors.

“The problem is that the health sector is very strong in convincing itself that other sectors should do something. And it is very weak in speaking the language of the other sectors… we work a lot on evidence, but as I said earlier, we usually frame it in a way to convince the health sector people, we don’t gather enough the evidence that is necessary to convince prime ministers, finance ministers, foreign ministers.” Interviewee 2

“You need to gear your stuff to the audience that you’re talking about. And again we’re not necessarily that adept at doing that. [There are] colleagues who kind of say, “We must do this purely from a public health point of view; they must realise that what we’re saying is important.”” Interviewee 3

The analysis of interviews indicates that WHO has ambitions to engage across sectors. However, staffing, organisational structure and financing factors serve to entrench the biomedical view of health despite widespread awareness of the linkages between health and its social, environmental and political determinants.

Firstly, it was noted that the current staffing of the organization, dominated by medical specialists, reinforces the dominant biomedical frame. The last report released by WHO on its staffing indicated that 47.8 % of the WHO staff had health professional background, of which 90.7 % were medical specialists, with 49.8 % of these being public health specialists. In comparison, with 0.1 % were economists and 1.6 % lawyers and social scientists [15, 30].

“I would say, you know, we are an organization, particularly at headquarters, that was set up to do technical work and we hire specialists. And we hire people with the right shaped heads to do detailed, technical work, but not with the right shaped heads to do policy and negotiation. I think that’s beginning to shift, but it’s a barrier” Interviewee 3

The second barrier is the structure of the organization and the resultant internal tensions. The specialized nature of WHO’s programmes and departments, and their often independent responsibility in fundraising and in implementing their mandate, leads to an overly narrow focus on specific health issues, priorities and interests. To this end, a range of conceptualizations of health exist between the different components, each of which seeks to improve public health in very different ways.

“I think one of WHO’s strengths, in a sense, lies much more in its programmatic structure and that dominates very much. We’re good at TB, we’re good at malaria, we’re good at health systems to some extent, we’re okay with all of that stuff. Where we are weak, if those are the pillars, the roof is very thin. The parts of the organisation that deal with health as a broader issue, that can have the breadth to look at a range of health issues, are few and far between…We somehow aren’t as good as we could be in terms of being greater than the sum of our parts.” Interviewee 3

“…as you go towards the more technical areas, then of course people who are working on diabetes are going to see things a little bit differently from people working on ebola…I think depending on the office that you’re operating in, what the predominant factors or pressures are, are going to differ a little bit. So there’s inevitably going to be a lot of different perspectives and views about what’s most important, what is the most pressing, but I think when you put it all together, probably the biggest thing is that there will be a fair amount of consistency in terms of what we are struggling with. I think the real difficulties are going to be over, what do you do about them?” Interviewee 5

It was argued that achieving alignment among different parts of the organization prior to engagement with other actors required negotiations between the various interests and perspectives of each department. Ultimately, this means that WHO often expresses views that are of the lowest common denominator–opinions that everyone can agree with—and are unable to prioritize between issues. As expressed by one interviewee:

“Trying to get a core script that people would be okay with, speaking in the language of the G8 rather than speaking in the language of having to cover every single WHO department was enormously hard. And usually you’re at greater risk, like in most wars, of being shot by your own side, of not including somebody’s pet priority, however irrelevant it might be.” Interviewee 3

A third reason for WHO remaining within a biomedical-oriented frame appears to be that the current financing of the organization does not incentivize WHO to staff and structure itself to engage with other sectors, and that member states are essentially compelling the agency to limit itself to being a technical agency through their financing power of individual programs focusing on diseases and specific health issues. It was expressed that member states are currently providing WHO with insufficient financial support for the organization to take on a coordinating role in global health.

“One of the barriers is also financial capacity…core funding has been capped since the 1980s and has therefore in real terms diminished. So at the same time, there is an expectation that we will do things [coordination], but we are actually not funded to do it… It’s difficult to coordinate without the funding for it.” Interviewee 1

“If WHO’s future is as a highly technical normative agency producing standards etc., then you need people with the in-depth specialist expertise to do that well. If WHO is going to be a political actor in the interests of health in other sectors, in international fora, you need people to do that. If it’s going to be a development player, you need to have people who can handle that stuff, but particularly at the developing country level. And at the minute, we talk about all 3, but at headquarters we’re still very much in Mode 1 because that’s where the money comes from, that’s what people want us to do.” Interviewee 3

It was explained that there is a split within the organization as well as a split in views among member states on whether to stay within the technical space or to aspire to a more proactive, political role where health is also advanced through intergovernmental negotiations.

“The question however becomes whether that trend of increasingly moving from technical strategies into inter-governmental negotiation continues and whether we put in place the capacity to manage it better or in effect devote more resources to it. And here I think within the organisation views are split. There are some that kind of [miss] the old days when it used to be a completely technical organization, and there are other that realised that this change is inevitable, and we’re one of the only organisations that can really put ourselves at the centre of these negotiations and we should therefore be quite happy to do more of them. And that split is reflected in member states as well.” Interviewee 3

It was suggested that even though ongoing WHO reforms mostly deal with managerial issues, it provides an opportunity for the organization to articulate how it could more effectively communicate and engage other sectors to achieve improved public health outcomes. However, instead of expanding its mandate, the organization had largely limited its operational focus to the health sector:

“Last year, we defined these six categories [for organizing WHO’s future work]. If you want my personal opinion, we missed a good opportunity to better express the importance of influencing other sectors…You will get something which is very medicalised and very health-care oriented, treating diseases, non-communicable, communicable diseases, life course, emergencies and health systems. Everything is very much around health care.” Interviewee 4

“…we need to convince our own peers as well, that universal health coverage is a beautiful thing, but that it is a health-related goal, where the health sector is responsible for the achievement. I will be very pleased if there are responsibilities all over the place [in the post-2015 agenda] for achieving health instead of just one.” Interviewee 4

This tension is reflected in WHO’s General Programme of Work. The previous programme of work (the 11th General Programme of Work (GPW11) [31] and the Medium-Term Strategic Plan 2009–2013 [32]), despite recognizing its inability to adequately address the broader determinants of health, only vaguely discussed how WHO should engage in cross-sectoral collaboration, largely limiting itself to being responsible for informing governments, but not demonstrating leadership (Table 4). There is however a visible shift in language between the 11th and 12th General Programmes of Work (GPW), with the latter more explicitly recognizing that the organization needs to put in greater capacity to manage inter-governmental processes as well as promote “health in a range of intergovernmental forums (foreign policy, trade negotiations, human rights, climate change agreements, and others) that do not have health as their prime concern, but whose decisions can have impact on health outcomes” [33]. However, it is noted that the largest emphasis in GPW12 is on universal health coverage as WHO’s most ambitious contribution–an issue that falls squarely within the health sector–with limited discussion about cross-cutting, underlying issues where sectors outside the health sector may participate in improving public health.

### WHO’s cross-sectoral engagement is hampered externally by siloed areas of focus for each IGO, and the lack of adequate conceptual frameworks and institutional mechanisms to facilitate engagement across siloes

While a broader approach to health requires collaboration and shared responsibility, interviewees recognized an inherent barrier in the way IGOs currently interact between sectors. Broadening WHO’s mandate to include and share leadership roles with other sectors was thought to risk diluting their mandate, potentially resulting in resources being shared or diverted away to other non-health actors. Similarly, two interviewees noted that WHO’s involvement in other sectors may be resisted by other IGOs that want to control their policymaking domains:

“…to be very honest, I think [barriers to collaboration between IGOs] are mandate, power, budget and fear. Fear in the sense that, well, ‘I don’t want to give [away] part of my organization’s mandate or power.” Interviewee 4

“Another level of issue of course is, who has the mandate to do something in an area? And particularly difficult is if it appears that you have overlapping mandates, so you have different organizations saying, “Well, we should be doing that.” And another organisation saying exactly the same thing. Then do you both repeat and do duplicative work, or do you try to reconcile somehow?” Interviewee 5

Despite expressing that the importance of cross-sectoral engagement has long been recognized, one interviewee argued that WHO, and the global health system more broadly, still mainly viewed health as an issue for the health sector.

“Unfortunately, the major actors in global governance might still feel when you talk about global governance for health, that this is the business of health actors. This is one of the major mistakes, in my opinion, to consider that health is the business of the health sector exclusively. This looks very old fashioned today, after years of talking about health in all policies and inter-sectoral work. I’m afraid it is one of the main mistakes, considering health to be about the health sector, about hospitals and health care.” Interviewee 4

The confluence of biomedical framing within the health sector and the siloing of actors across sectors appeared to have implications for the type of collaborative partnerships that deal with health in the global governance system. Here, the H8 group^1^ was used as an example of an initiative which has evolved around a narrowly conceived mandate.

“If you look at all of them [the members of the H8 group], they are very much health-related. They consider themselves as very much the core of the health business... They think that they are doing a lot in terms of primary prevention because they go as far as vaccination. With all due respect, the H8 is a very important and influential group, but the mistake is to keep the boundaries on that group around health-specific issues, with health actors and ministers of health essentially as the main interlocutors….So I think this is the major issue of the moment, that health remains very much around health issues, in the old fashioned way of considering health of people.” Interviewee 4

The recent UN reform effort (“Delivering as One”) was presented as a way of dealing with multiple mandates, but it was felt that in reality, it only added to the complexity, ultimately encouraging individual UN agencies to maximize their access to resources.

“It (UN Delivering as One) is sort of pious words and an awful lot of process, whilst everybody is elbowing others out of the way to get hold of the resources.” Interviewee 3

“If you would allow me to present a negative example. The UN decided to look at ‘Delivering as One’, as part of its strategic thinking. Instead of having so many agencies, it decided to have a culture of ‘delivering as one’. What has happened is now we have all the specialist agencies, plus one called ‘UN Delivering as One’. This is the proof that we already have failed.” Interviewee 4

A number of examples of previous and existing collaborations between WHO and other IGOs and non-health groups were identified through the document review, demonstrating that WHO does indeed seek engagement with other sectors (Table 5). It was beyond the scope of this study to evaluate the effectiveness of the inter-institutional mechanisms that have been implemented, and the cross-sectoral work performed by the WHO. However, three important features were noted. One was that the agreements between WHO and other IGOs appeared, where possible, to demarcate areas of primary responsibility. Second, it was noted that these collaborations appear to not have resulted in formal platforms or institutional mechanisms for cross-sectoral dialogue, but rather relied on governing bodies and formal meetings of each, respective organization. Finally, the annual updates on global collaboration with organizations in other sectors appeared to indicate a reduction in the number of collaborative activities during the latter part of the recent decade. However, the review of WHO’s annual updates to the governing bodies gives an incomplete picture, since individual WHO departments also pursue cross-sectoral collaborations beyond those formally reported to the governing bodies. A longstanding collaboration between the WHO and the FAO is the Codex Alimentarius Commission, which issues standards and codes of practice related to foods, food production, and food safety, and which is recognized by the World Trade Organization (WTO) as an international reference point for the resolution of disputes concerning food safety and consumer protection. Other examples of cross-sectoral collaboration include the tri-lateral cooperation on intellectual property and public health between WHO, World Intellectual Property Organization and WTO [34], the recently established WMO-WHO joint office for climate and health [35], and the planned collaboration between the WHO, the UNDP, the World Bank and other IGOs on the prevention and control of non-communicable diseases [36, 37]. The WHO have also the past 15 years at different times attempted to indirectly engage other sectors in the global health agenda by establishing commissions addressing specific issues which require cross-sectoral collaboration, namely the Commission on Macroeconomics and Health [38], the Commission on Intellectual Property Rights, Innovation and Public Health [39], the Commission on Social Determinants of Health [5], and now more recently the Commission On Ending Childhood Obesity [40], which at the time of writing is yet to release its final report.

**Table 5:** Examples of cross-sectoral collaboration between the WHO and other IGOs reported in annual updates to the governing bodies

Organization	Example of collaboration^a,b,c^
World Trade Organization (WTO)	2001 • Joint WHO/WTO meeting on differential drug pricing and financing of essential drugs in April 2001 [46] 2002 • WTO contributed with research to a report prepared for the WHO on the links between tobacco consumption and trade liberalization in 2001 [47] • Joint WHO/WTO study on the implications of international trade and multilateral trade agreements for health systems and health service provision [48]
International Labour Organization (ILO)	1998 • Collaboration sought within the framework of the WHO Global Strategy for Occupational Health for All, to increase priority to occupational health and safety on national and international agendas 2000 • Agreed in 1999 to establish an inter-secretariat working group in order to promote cooperation in areas such as poverty alleviation, gender issues in workers’ health, prevention and control of HIV/AIDS among workers, and health financing and health insurance coverage for workers. 2010 • WHO and ILO were designated lead agencies for a UN system initiative on social protection (one of nine initiatives to help member states respond to the economic crisis)
International Monetary Fund (IMF)	2000 • WHO, IMF and the World Bank continued discussions begun in 1998 on health policies and measurement of health-system performance. • IMF participated in the policy advisory group of the Tobacco Free Initiative and in the Commission on Macroeconomics and Health.
World Bank	1998 • The World Bank adopted the policy of WHO partnership for health development, including collaboration at country level where WHO’s technical expertise is mobilized to improve the design, supervision and evaluation of World Bank-supported projects, and global collaboration where the WHO and the World Bank together advance international understanding of health, nutrition and population issues. 1999 • The World Bank joined WHO and other organizations in the partnership for Roll Back Malaria and the Tobacco Free Initiative. Previously, the World Bank had co-sponsored the Programme of Research, Development and Research Training in Human Reproduction in 1988, and the Special Programme for Research and Training in Tropical Diseases from the start in 1975. 2000 • Poverty reduction identified as an important area for collaboration • WHO’s policy guidance considered to be helpful for shaping the design of health and social development projects financed by the World Bank. • The World Bank agreed to co-sponsor the Global Alliance for Vaccines and Immunization. 2001 • WHO cooperated on poverty reduction in the context of the Heavily-Indebted Poor Countries (HIPC) initiative. Within the framework of HIPC, WHO and UNICEF collaborated with the World Bank on the health, nutrition and population components of the poverty reduction strategy papers. • The World Bank and WHO worked together on health systems and health care financing, and prepared together with ILO a paper for WHO’s Commission on Macroeconomics and Health. 2002 • WHO analysed health in the strategy papers for 10 countries presented at the IMF/World Bank International Conference on Poverty Reduction Strategies 2003 • The World Bank, as part of its initiative to accelerate progress towards the health-related goals, convened organizations in the United Nations system, including WHO, and donors to examine approaches to scaling up activities. WHO’s main role was reported to be addressing cross-cutting issues influencing achievement of goals, such as those related to human resources, governance and human rights. 2004 • WHO collaborated with the “anchor unit” of the World Bank’s Human Development Network to promote deworming activities in the FRESH Start initiative (Focusing Resources on Effective School Health).
Food and Agriculture Organization of the UN (FAO)	1998 • Close collaboration in support of the World Declaration and Plan of Action for Nutrition, and the country implementation of over 160 national food and nutrition policies and plans of action. • WHO involved in the Inter-Agency Working Group on Food Insecurity and Vulnerability Information and Mapping System as part of global follow-up to the World Food Summit of 1996. • WHO and FAO facilitated the work of the Codex Alimentarius Commission (which issue standards, recommendations and guidelines explicitly recognized in the World Trade Organization Agreement on the Application of Sanitary and Phytosanitary Measures as the international reference for food safety). 2001 • In the context of long-standing collaboration with FAO within the Joint FAO/WHO Food Standards Programme and joint expert committees, WHO was active on standards for infant and young child feeding, and on salt iodination. The two organizations also intensified efforts to develop an information and mapping system on food insecurity, vulnerability and poverty. 2003 • The Report of the Joint WHO/FAO Expert Consultation on Diet, Nutrition and the Prevention of Chronic Diseases [49] provided scientific foundation for WHO’s elaboration of a global strategy on diet, physical activity and health
World Food Programme (WFP)	2000 • WFP a partner in training programmes for medical doctors, midwives, nurses and others involved in implementing WFP’s food assistance projects. 2007 • Collaboration agreement signed between WFP and WHO, as part of reform in the area of humanitarian assistance, logistics planning and implementation, whereby WHO will coordinate health logistics through the WFP network of humanitarian response depots and WFP will provide priority logistical services to WHO’s human and technical resources during emergencies.
UNESCO	1998 • Cooperation geared to promoting health of school-age children and young people, adult education, and physical activity for health. • UNESCO collaborated actively with WHO and other concerned partners in the launch and worldwide promotion of the Global Initiative on Active Living/Physical Activity for Health. 2000 • In September 1999, WHO led an interagency discussion with the World Bank, UNESCO and UNICEF on the development of a common agenda on school health programmes, which resulted in the Focusing Resources on Effective School Health (FRESH) approach, later launched during the World Education Forum in Dakar in 2000. The FRESH framework was designed to capture education, health, nutrition and overall development goals and provides the necessary intersectoral institutional support to ensure sustainability [50]. • UNESCO and WHO cooperated to support skilled-based health education for: HIV prevention; tobacco use prevention; health and nutrition; violence prevention; and caring for the environment. This collaboration generated methodological tools and age-appropriate material for teachers in developing countries to use in primary and secondary schools.
International Atomic Energy Agency (IAEA)	1998 • WHO facilitated, together with the IAEA and FAO, the work of the International Consultative Group on Food Irradiation, an intergovernmental body with membership of 47 countries and convened a study group on high dose food irradiation. 2002 • Technical consultations conducted to streamline collaboration, covering areas such as radiotherapy, diagnostic procedures, molecular biology, communicable diseases, food safety and nutrition, and health-related aspects of radiation protection. 2003 • Started collaboration on building human resource and institutional capacity for the application of telecommunications to the maintenance of nuclear medicine equipment in developing countries.
World Organisation for Animal Health (OIE)	2007 • The Global Early Warning and Response System launched by the FAO, OIE and the WHO, as the first joint early warning and response system for animal diseases, including zoonoses, to enhance global capacity to detect and control diseases of animal origin at their source.
UNCTAD	1998 • Explored the issue of trade in health services, and issued a joint publication which examined trade and health implications, especially from the developing country perspective [51]. • Collaborated on building up country capacity to analyse and respond to the effects of globalization and trade on health, and on a framework for integrating health protection into UNCTAD’s plan of action.

^a^Collaborations may have been ongoing for several years before being reported to the governing bodies, and also continued without the governing bodies receiving further updates

^b^Collaborations at the regional and national level is not covered by this overview

^c^Collaborations on organizing international conferences has not been included in this overview

Several interviewees suggested that very few effective institutional mechanisms exist for improving the interaction between IGOs of different sectors. A number of policy frameworks seek to frame health broadly, and integrate a concern for health within other sectors. One such prominent platform is the social determinants of health framework, which provides a foundation for understanding the relationship between individual health and broader social and environmental conditions. The ‘Health in All Policies’ approach is a similar concept, which aligns naturally with this framework. However, some interviewees believed that these approaches, despite being talked about for many years, have largely remained ‘health-centric’, and have been insufficient for engaging other sectors.

“And then obviously all the work on the social determinants of health, where I would say conceptually we have advanced quite well, while the practical tools of how we actually do it we haven’t progressed enough. Part of it is linked to the fact that doctors are trained to prescribe, and the social determinants of health, or if you take concepts like health-in-all-policies, are basically still health-centric, and haven’t shifted to whole-of-government, whole-of-society approaches, delivering all key goods and commodities, including health, that society needs.” Interviewee 2

“I think there’s a lot of talk and not much action about multi-sectoral this and that. We’ve been talking about it for [so many] years, but actually a good analysis of how you drive policy change across [all sectors of] government, let alone across societies, is extremely difficult to do, particularly if you’re obviously trying to do it in the interests of better health. And the recent enthusiasm for social determinants as a way of thinking about exactly the same problem, adds a new set of vocabulary without really adding, in my view, convincing institutional mechanisms for actually doing the business.” Interviewee 3

Expanding an IGO’s operations and achieving coherence with other sectors can be trying, as it brings with it a number of complex and poorly understood organizational management challenges. As suggested by one interviewee, these challenges should be recognized as common to all UN agencies and government departments, and not unique to WHO.

“So WHO is a complexity in itself, which struggles like any other organisation, not more than others, but like any other organisation with policy coherence. It’s the same question if you ask, what’s a country’s opinion on this, and you ask the same question to different ministries, the normal is that you get different answers.” Interviewee 2

## Discussion

The findings from this study suggest that WHO’s operations currently centre upon a narrow, biomedical view of health rather than a view which incorporates the broader determinants of human wellbeing. There is a shared understanding that the dominance of the biomedical approach is an impediment to WHO playing an effective cross-sectoral coordinating role for health. The interviewees and documents reviewed suggest a number of reasons for this approach, which can be divided into both internal and external pressures.

The language used both by interviewees and WHO’s public documents portrays the sense of a constrained organization which is forced to discuss health as though it is a series of discrete issues which can be dealt with independently, even though the organization’s leadership is aware that the reality is far more nuanced. The biomedical approach is reflected in WHO’s programme of work, and underpinned by budgetary allocations which are primarily structured around discrete disease and treatment groups. Importantly, this study suggests that external political pressures and the lack of financial flexibility afforded by member states constrain WHO, and contribute to maintaining a narrow technical focus which impedes cross-sectoral collaboration.

A number of global health experts have drawn attention to the state of WHO’s budget, 80 % of which consists of voluntary contributions earmarked for a specific purpose, and to how this lack of discretionary budget constrains the organization’s ability to implement the programme of work approved by the World Health Assembly [16, 41–44]. The programme budget for 2014–15 allocates more resources to specific disease-based efforts compared to more broadly defined programmatic areas addressing social and environmental determinants of health (($841 million allocated to communicable diseases and §318 million for non-communicable diseases compared to $28 million and $91 million respectively for social determinants of health, and health and the environment) [45].

The organization’s dominant biomedical approach to its mandate is also likely influenced by the dominance of medical professionals [30], who may have excellent technical knowledge of health issues, but not the necessary expertise to support political negotiations between member states, which require skills in convening negotiations and facilitating consensus-building [6, 15]. The lack of staff with other backgrounds may also limit the organization’s capacity to advocate for health in other sectors such as trade, environment or labour and migration. The need to review WHO’s staffing was noted in a recent Chatham House on International Affairs’ analysis of the organization, which argued that “addressing the social, economic and environmental determinants of health and non-communicable disease, and advising countries on the attainment of universal health coverage and financial protection would seem to demand a very different distribution of skills from that which exists currently” [14]. There is likely also a complex interaction between the external image portrayed by WHO as a ‘doctor of the world’ (which is undoubtedly influenced by the external pressures noted above) and the medicalization of its internal organizational culture.

Externally, the lack of effective institutional mechanisms to facilitate effective cross-sectoral collaboration is a critical barrier. Interviewees suggested that existing frameworks for understanding the interaction between public health and other sectors (such as the social determinants of health) and designing interventions to engaging sectors in improving public health (such as the Health-in-All-Policies Approach) currently are alone insufficient for enabling dialogue and collaboration between different sectors affecting global health. Furthermore, interviewees noted pressures between different UN bodies, which ensure they stick firmly to their ‘space’, even when they recognize the need for shared responsibility to fulfill their mandates. One such pressure–in simplified terms–may follow the logic that ‘if WHO is telling us that major determinants of global health are sustainability and stable employment, perhaps we should fund UNEP and ILO to achieve improved health’. There is hence a perceived danger in stepping too far away from the biomedical understanding of health, in that WHO may cede power and resources to other IGOs. This concern extends internally to relationships between clusters and departments within WHO due to competition for funds and resources, and indeed functions at a national level between government departments.

It is important to note that these pressures are not unique to WHO. Crucially, these pressures and challenges–of trying to work across sectors to enhance human wellbeing in an increasingly complex world–are in no way unique to multilateral organizations. They are common issues that national governments, civil society groups, and businesses face on a regular basis. Indeed, despite the predominance of the biomedical frame throughout WHO, it appears that the organization’s senior leadership has a firm understanding and vision of how they believe the agency ought to collaborate and engage in cross-sectoral collaboration, within the boundaries set by its constitution. Given the pressures outlined above, a disconnect between the culture, competence, activities and public perception of WHO and its senior leaders is to be expected.

There are three main limitations to this study. First, its conclusions are based on a limited number of interviews, and the possibility exists that important perspectives may have been missed, which could have been captured by a larger sample. This aspect is partially addressed by the purposive sampling of interviewees with a high degree of seniority and leadership experience in the WHO, giving them a firm understanding of the organization’s strategic direction, organizational culture, and the various challenges experienced in managing its programming. Second, it is well known that global governance for health involves the interaction of many actors, including governments, civil society, businesses, and public-private partnerships–bias may result from seeking perspectives from only one of these potential stakeholders. Third, as the study is specific to WHO and its current situation, generalisation of results to other organizations or sectors may be inappropriate.

## Conclusion

A changing global environment is placing new and complex demands on the UN System, including WHO. These require a broader approach to promoting human health and wellbeing, which is conceptualized in WHO’s constitution and well understood by senior WHO officials, but has yet to be sufficiently implemented. In contrast, there are a number of external and internal pressures on the organization which have created an organizational culture and operational structure which focuses on a narrow, technical approach to global health, prioritizing disease-based, siloed interventions over cross-sectoral collaboration. Furthermore, conceptual frameworks such as the social determinants of health framework and Health-in-All-Policies are not enough. Member states must incentivize and support inter-governmental organizations in pursuing collaboration. There is also a need for fora, platforms and institutional mechanisms to facilitate cross-institutional discussions and negotiations. New forms of operationalizing and enhancing cross-sectoral partnerships must become a priority for everybody seeking to improve global governance for health.

## Additional files

Additional file 1:
**List of documents reviewed for the WHO documentary review.** (DOCX 45 kb) Additional file 2:
**Data collection matrix used for IGO documentary review.** (DOCX 29 kb)

## Footnotes

1 The Health 8 group is an informal coalition of organizations made up of WHO, UNICEF, UNFPA, UNAIDS, the Global Fund, GAVI, and the Bill and Melinda Gates Foundation
