# Supplementary material for: Conceptual and institutional gaps: understanding how the WHO can become a more effective cross-sectoral collaborator
Source: Global Health. 2015 Nov 24;11:46. doi: 10.1186/s12992-015-0128-6 (PMC4657201; doi:10.1186/s12992-015-0128-6)
Supplement: Additional file 1: — List of documents reviewed for the WHO documentary review. (DOCX 45 kb) [file 12992_2015_128_MOESM1_ESM.docx]

**Additional file 1 – List of documents reviewed for the WHO documentary review**

1. WHO. Twelfth General Programme of Work 2014-2019. Not merely the absence of disease. Geneva, Switzerland: World Health Organization; 2014.
2. WHO. Engaging for Health.11th General Programme of Work, 2006-2015. A Global Health Agenda. Geneva, Switzerland; 2006.
3. WHO. Medium-term strategic plan 2008-2013. Interim assessment. Geneva, Switzerland; 2011.
4. World Health Organization. Progress report on reform implementation. Report by the Director-General. Geneva: World Health Organization; 2014.
5. WHO. Appendix 3 - Supporting documents to the financial dialogue. Approved Programme Budget 2014-15. Geneva, Switzerland; 2013.
6. WHO. Proposed programme budget 2014-2015. Geneva, Switzerland: World Health Organization; 2013.
7. World Health Organization. Human resources: annual report. Report by the Secretariat. [Internet]. 2013 [cited 2014 Nov 1]. Available from: <http://apps.who.int/gb/ebwha/pdf_files/WHA66/A66_36-en.pdf>
8. WHO. WHO’s role in global health governance. Report by the Director-General. Geneva, Switzerland; 2013.
9. World Health Organization. Progress report on reform implementation. Report by the Director-General. Geneva: World Health Organization; 2014.
10. WHO. Terms of reference for the UN interagency task force on the prevention and control of noncommunicable diseases. Geneva, Switzerland; 2015.
11. PricewaterhouseCoopers SA. WHO reform. Stage 2 evaluation. Final Report. Geneva, Switzerland; 2013.
12. PricewaterhouseCoopers SA. WHO Financing Dialogue. Final report. Geneva, Switzerland; 2014.
13. WHO. Collaboration within the United Nations system and with other intergovernmental organizations. Report by the Secretariat. Geneva, Switzerland; 1999.
14. WHO. Collaboration within the United Nations system and with other intergovernmental organizations. Report by the Secretariat. Geneva, Switzerland; 2000.
15. WHO. Collaboration within the United Nations system and with other intergovernmental organizations. Report by the Secretariat. Geneva, Switzerland; 2001.
16. WHO. Collaboration within the United Nations system and with other intergovernmental organizations. Report by the Secretariat. Geneva, Switzerland; 2002.
17. WHO. Collaboration within the United Nations system and with other intergovernmental organizations. International Decade of the World’s Indigenous People. Report by the Secretariat. Geneva, Switzerland; 2002.
18. WHO. Collaboration within the United Nations system and with other intergovernmental organizations. Report by the Secretariat. Geneva, Switzerland; 2003.
19. WHO. Collaboration within the United Nations system and with other intergovernmental organizations. Report by the Secretariat. Geneva, Switzerland; 2004.
20. WHO. Collaboration within the United Nations system and with other intergovernmental organizations. Report by the Secretariat. Geneva, Switzerland; 2005.
21. WHO. Collaboration within the United Nations system and with other intergovernmental organizations. Report by the Secretariat. Geneva, Switzerland; 2006.
22. WHO. Collaboration within the United Nations system and with other intergovernmental organizations. Report by the Secretariat. Geneva, Switzerland; 2007.
23. WHO. Collaboration within the United Nations system and with other intergovernmental organizations. Piloting the One UN Country Programme in eight countries. Report by the Secretariat. Geneva, Switzerland; 2007.
24. WHO. Collaboration within the United Nations system and with other intergovernmental organizations. Report by the Secretariat. Geneva, Switzerland; 2008.
25. WHO. Collaboration within the United Nations system and with other intergovernmental organizations. Report by the Secretariat. Geneva, Switzerland; 2009.
26. WHO. Collaboration within the United Nations system and with other intergovernmental organizations. Report by the Secretariat. Geneva, Switzerland; 2010.
27. WHO. Collaboration within the United Nations system and with other intergovernmental organizations. Report by the Secretariat. Geneva, Switzerland; 2011.
28. WHO. Collaboration within the United Nations system and with other intergovernmental organizations. Report by the Secretariat. Geneva, Switzerland; 2012.
29. WHO. Collaboration within the United Nations system and with other intergovernmental organizations. Report by the Secretariat. Geneva, Switzerland; 2013.
30. WHO. Collaboration within the United Nations system and with other intergovernmental organizations. Report by the Secretariat. Geneva, Switzerland; 2014.
31. WHO. Agreements with other intergovernmental organizations [Internet]. Geneva, Switzerland; 2011 [cited 2015 Apr 1]. Available from: http://apps.who.int/gb/bd/PDF/bd47/EN/agreements-with-other-inter-en.pdf
