# Supplementary material for: Conceptual and institutional gaps: understanding how the WHO can become a more effective cross-sectoral collaborator
Source: Global Health. 2015 Nov 24;11:46. doi: 10.1186/s12992-015-0128-6 (PMC4657201; doi:10.1186/s12992-015-0128-6)
Supplement: Additional file 2: — Data collection matrix used for IGO documentary review. (DOCX 29 kb) [file 12992_2015_128_MOESM2_ESM.docx]

**Additional file 2 – Data collection matrix used for IGO documentary review**

| **IGO data collection matrix** |
| --- |

### Baseline information

**General**

1. **Organisational structure and governance**
2. **Stated objectives of the organisation**

**Questions relevant for non-health IGOs**

1. **Areas/branches of IGO directly relevant to health**
2. **Financial resources spent on advancing global health**
3. **Areas of collaboration with Ministries of Health/health NGO's/healthcare organisations/the WHO**

| Matrix for collecting statements framing global health from strategic documents of IGOs | |
| --- | --- |
| Frames | **Findings** |
| **Global health as biomedicine**  “At its core, EBM encourages and reinforces  positivist, rationalist ways of reasoning _ namely, that a world exists independent of  observation that can be analysed using epidemiological and biostatistical tools to  provide data that will inform health-related policy decisions. As a direct result of its integration into contemporary training programmes, successive generations of medical/clinical practitioners have been trained in EBM methods and ways of thinking. As a result, EBM has become the primary mode of scientific, rational enquiry for contemporary biomedicine and clinical practice and the key frame for the  health policy community” (McInnes et al, 2012)  **“**Global health as public health,  seeks to decrease the worldwide burden of disease, with priority given to those risk factors and diseases  that make the greatest contribution to this burden. Resources will be directed to maximise the potential  health eff ects.” (Stuckler & McKee, 2008) |  |
| **Global health as a commodity/trade issue**  ***“****Global public goods discourse must compete in treasury departments with the one outlier – health as tradable good. There is some pretence that such trade will lead to better outcomes, but the reality is that health is reduced to goods (such as drugs and new technologies) or services (private health insurance, facilities or providers), the increased*  *cross-border flow of which is designed to maximise profit, not health.”* *(Labontè, 2008)* |  |
| **Global health as foreign policy (“global health diplomacy”)**  ”based on politicians using global-health policies toc reate a positive worldwide reputation and exert political  influence, forging alliances with countries where they have strategic interests, opening new markets for trade,  and protecting domestic pharmaceutical companies. Global-health priorities follow foreign-policy goals.” (Stucklet & McKee, 2008)  “No integrating discourse as yet exists. There are some movements in that direction. One that holds potential is global health diplomacy, in which the ethical, rights-based, public goods, development and security arguments are all brought to bear on why improving global health equity should be a central plank in governments’ foreign policy.” (Labontè, 2008) |  |
| Global health as a global public good  ***“****Global public goods provides a language by which economists of one market persuasion can convince economists of another that there is a sound rationale for a system of shared global financing and regulation.”* *(Labontè, 2008)* |  |
| **Global health as a human right**  “Over the past 20 years, there has been a marked resurgence in framing global  public health issues in terms of human rights…human rights are moral values that should guide public health experts and  ensure that their policies and practices are not discriminatory, coercive or undemocratic. Another influential understanding of human  rights and health is that which developed during the twenty-first century by both the  UN Committee on Economic, Social and Cultural Rights (UNCESCR) and the UN  Special Rapporteur on the Right to Health, Paul Hunt. For them, the relationship  between human rights and health is primarily about the right to health: the right to receive appropriate and affordable health care” (McInnes et al, 2012)  “Human rights, though weak in global enforcement, has advocacy traction and legal potential within national boundaries. Such rights do not resolve embedded tensions between the individual and the collective, an issue to which human rights experts are now  ***attending****.”* **(Labontè, 2008)** |  |
| **Global health as investing in economic development**  *”Global health as investment involves the use of health as a means of maximising economic development,*  *a view exemplified by WHO’s Commission on Macroeconomics and Health. The focus is on young and working-age people, and on diseases seen as acting as a brake on development, such as AIDS, tuberculosis, and malaria, as well as veterinary diseases of economic*  *importance.The final metaphor, global health as public health,*  *seeks to decrease the worldwide burden of disease, with priority given to those risk factors and diseases*  *that make the greatest contribution to this burden. Resources will be directed to maximise the potential*  *health eff ects.”* (Stuckler & McKee, 2008)  *“Development remains the invitation to global governance debates. It provides a seat at the table. Risks inherent in its ‘investing in health’ instrumentalism can be tempered by*  *continuously reminding decision makers to distinguish ‘which one is the objective (human development) and which one the tool (economic growth)’ (Global Forum for Health Research 2004). The accountability advocacy of international NGOs continues to pressure rich nations to move beyond the inadequate patchwork of broken aid promises to a global system of taxation and redistribution.” (Labontè, 2008)* |  |
| **Global health as a mean to reduce poverty**  **“**Global health as charity involves the promotion of health as a key element in the fight against poverty. Priorities are often indicative of popular views of victimhood, so the benefi ciaries are those seen as most deserving by those who must contribute to their relief. Consequently, the focus is typically on mothers and children, and on issues such as malnutrition, natural  disasters, and safe childbirth.” (Stuckler & McKeen, 2008) |  |

| **Global health as a security issue**  ”Where health policy seeks to protect one’s own population, focusing mainly on communicable diseases that threaten this population. Only diseases of poor countries that pose a potential threat to citizens of rich countries matter. Thus, diseases such as severe acute respiratory syndrome, avian infl uenza, and drug-resistant tuberculosis are prioritised,9 whereas leprosy, filariasis, and schistosomiasis are deprioritised. Health policy is integrated with protection from  bioterrorism, missile shields, and “defensive” warfare.” (Stuckler & McKee, 2008)  **“**Thus, health becomes a security issue when it is perceived and presented in the following ways: (1) as a threat to someone or something and (2) as something which defensive measures (either in the form of  prevention or response) must be taken against. This is the hallmark of the security frame in global health: x is a threat/risk to a referent object in respect to which we must put defensive/protective measures y in place.” (McInnes et al, 2012) |  |
| --- | --- |
